# Reevaluation des Notarzteinsatzindikationskataloges nach Verkehrsunfällen

**DOI:** 10.1007/s00113-023-01408-8

**Published:** 2024-02-02

**Authors:** Michael Hetz, Stefan Babisch, Thomas Unger, Klaus-Dieter Schaser, Christian Kleber

**Affiliations:** 1 Klinik und Poliklinik für Orthopädie, Unfallchirurgie und Plastische Chirurgie (OUP), Department für Operative Medizin (DOPM), Universitätsklinikum Leipzig (AöR), Liebigstraße 20, 04103 Leipzig, Deutschland; 2 Verkehrsunfallforschung an der Technischen Universität Dresden GmbH, Semperstr. 2a, 01069 Dresden, Deutschland; 3 UniversitätsCentrum für Orthopädie, Unfallchirurgie und Plastische Chirurgie, Universitätsklinikum Carl Gustav Carus Dresden, Fetscherstraße 74, 01307 Dresden, Deutschland

**Keywords:** Unfalldatenanalyse, Rettungsdienstoptimierung, Unfallparameter, Fahrzeugsicherheit, Trauma-Registeranalyse, Accident data analysis, Rescue service optimization, Accident parameters, Vehicle safety, Trauma register analysis

## Abstract

**Hintergrund:**

Der Notarztindikationskatalog basiert auf veralteten Studien und gibt wenig Anhalt für Alarmierungskriterien nach Verkehrsunfällen. Fortschritte der Fahrzeugsicherheitstechnik und Veränderungen der verfügbaren Ressourcen machen eine Reevaluation der Indikationen notwendig. Ziel dieser retrospektiven Registerstudie ist die Identifizierung von präklinisch erfassbaren Unfallvariablen für schwere Verletzungen nach Verkehrsunfällen.

**Methodik:**

Im Zeitraum 01.01.2000–31.12.2021 wurden 47.145 Verunfallte anhand der GIDAS-Datenbank eingeschlossen. Separate Datensätze für Schwer- (AIS 3+) und Leichtverletzte wurden ausgewertet.

**Ergebnisse:**

Herausschleudern (PPW 80,6 %), Einklemmung (PPW 75,6 %), brennende Fahrzeuge (PPW 57,1 %), problematische Rettung (PPW 56,3 %), Fahrzeugzerreißung (PPW 51,6 %) und Amnesie (PPW 50,3 %) wiesen auf schwere Verletzungen bei Fahrzeuginsassen hin. Bei ungeschützten Verkehrsteilnehmern (Motorrad‑, Fahrradfahrende, zu Fuß Gehende) wurden auch Helmverlust (PPW 61,1 %), Überrollen/Mitschleifen (PPW 41,9 %), Scheibenbruch am Gegnerfahrzeug (PPW 35,8 %) und Folgeanprall mit Objekten (PPW 31,1 %) identifiziert. Der Chi-Quadrat-Test zeigte signifikante Assoziationen zwischen den Variablen und schweren Verletzungen. Kombinationsvariablen erreichten PPW-Werte über 82 %.

**Diskussion:**

Der Notarztindikationskatalog liefert kaum präklinisch feststellbare Kriterien und sollte anhand der objektiven Registerdaten überarbeitet werden. Abfragemodelle für Leitstellendisponenten sollten getestet werden.

**Zusatzmaterial online:**

Die Online-Version dieses Beitrags (10.1007/s00113-023-01408-8) enthält eine Tabelle mit den Bedeutungen der GIDAS-Variablen.

## Abstract (en)

**Background:**

The emergency physician indication catalogue is based on outdated studies and provides limited guidance for alarm criteria following traffic accidents. Advances in vehicle safety technology and changes in available resources necessitate a re-evaluation of the indications. The aim of this retrospective registry study is to identify preclinically assessable variables for severe injuries sustained in traffic accidents.

**Methods:**

A total of 47,145 individuals involved in accidents between 1 January 2000 and 31 December 2021 from the GIDAS database were included. Separate datasets for severe (AIS 3+) and minor injuries were evaluated.

**Results:**

Ejection (PPV 80.6%), entrapment (PPV 75.6%), burning vehicles (PPV 57.1%), challenging rescue situations (PPV 56.3%), vehicle disintegration (PPV 51.6%), and amnesia (PPV 50.3%) indicated severe injuries among vehicle occupants. For vulnerable road users (motorcyclists, cyclists, pedestrians), helmet loss (PPV 61.1%), being run over/dragging (PPV 41.9%), opponent vehicle window breakage (PPV 35.8%), and subsequent collision with objects (PPV 31.1%) were also identified. The χ^2^-test revealed significant associations between the variables and severe injuries. Combined variables achieved PPV values above 82%.

**Discussion:**

The current emergency physician indication catalogue provides limited preclinically detectable criteria and should be revised based on the objective registry data. Query models for emergency dispatchers should be tested.

**Graphic abstract:**

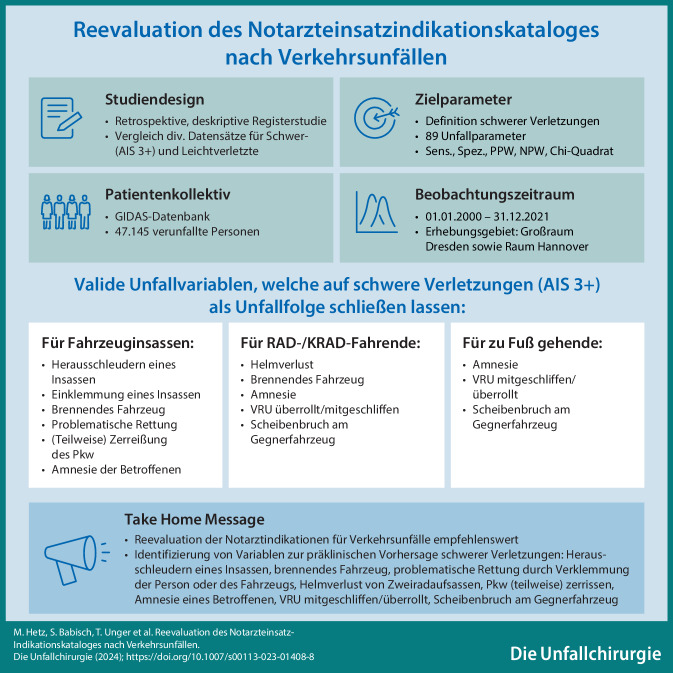

Der Notarztindikationskatalog für Verkehrsunfälle basiert auf veralteten Studien. Fortschritte in der Fahrzeugsicherheit erfordern eine Neubewertung, um Fehlalarmierungen zu reduzieren und die Versorgung zu gewährleisten. Diese Studie identifiziert präklinisch für Laien erfassbare Variablen zur Vorhersage schwerer Verletzungen nach Verkehrsunfällen. Mit über 28 Variablen und deren Kombinationen konnten valide Vorhersagewerte erreicht werden. Basierend auf objektiven Registerdaten empfehlen wir die Reevaluation des Notarztindikationskataloges.

## Hintergrund und Fragestellung

Die Notarztalarmierung nach einem Verkehrsunfall (VKU) erfolgt anhand von Empfehlungen des Notarztindikationskatalogs (NAIK) der Bundesärztekammer (BÄK) [1]. Die aktuelle Version des NAIK wurde 2013 beschlossen und beruht auf dem NAIK aus dem Jahr 2001 [1]. Sowohl die Art der Verkehrsteilnahme [2, 3] als auch die Verkehrssicherheitssysteme [4] haben sich seither deutlich verändert. Daher ist eine Reevaluation der Variablen zur Vermeidung von Fehleinsätzen notwendig und sinnvoll.

Eine umfangreiche Quelle technischer und medizinischer Daten nach Verkehrsunfällen stellt die Datenbank der German In Depth Accident Study (GIDAS) dar. Diese wird kontinuierlich mit Verkehrsunfalldaten gespeist, welche direkt an der Unfallstelle erhoben werden. Die GIDAS-Variablen umfassen sowohl medizinische Aspekte (z. B. Bewusstseinslage der Betroffenen) als auch umfangreiche technische Daten und Unfallmodalitäten (Tageszeit, Ortslage, Kollisionsgeschwindigkeiten etc.). Ziel ist es, anhand dieser Daten Variablen zu identifizieren, welche zu schweren Verletzungen führen und damit einem Notarzteinsatz indizieren sollten. Eine Notarztalarmierung erfolgt, nach aktuellen NAIK, bei „Verdacht auf fehlende oder deutlich beeinträchtigte Vitalfunktion“, nach „schwerem Verkehrsunfall mit Hinweis auf Verletzte“ bzw. „sonstigem Unfall mit Schwerverletzten“ [1]. Konkrete Unfallkonstellationen werden, abgesehen von Brand und Einklemmung, bisher nicht genannt.

Vor dem Hintergrund der geplanten Überarbeitung des Notarzteinsatzindikationskataloges der Bundesärztekammer, steigender Alarmierungszahlen, einer gebietsweise kaum noch sicherzustellenden Abdeckung in der notärztlichen Versorgung [5, 6] und häufigen Ambulantisierungen vom Patient*innen nach einem VKU trotz Schockraumalarmierung in den Kliniken wurden in dieser Arbeit zunächst „schwere Verkehrsunfälle/Verletzungen“ definiert. Nach Berechnung des positiven prädiktiven Wertes (PPW) sollen valide, präklinisch für Laien einfach erhebbare Variablen identifiziert werden, welche schwere Verletzungen nach einem VKU wahrscheinlich machen.

## Studiendesign und Untersuchungsmethoden

### Datengrundlage

Datengrundlage für diese deskriptive, retrospektive Registerstudie stellt die GIDAS-Datenbank (Abzug vom 31.12.2021; Erhebungsgebiete: Großraum Dresden sowie bis Ende 2019 Raum Hannover) dar. Die verwendeten Codes der Abbreviated Injury Scale (AIS) zur Verletzungskodierung basieren auf der Revision von 2015. Eingeschlossen wurden im Erhebungszeitraum 01.01.2000–31.12.2021 Verkehrsunfälle mit Personenschaden, deren technische Unfallrekonstruktionen vollständig vorlagen. Ausgeschlossen wurden Unverletzte oder Fälle mit unvollständig bearbeiteten Datensätzen.

### Definition schwere Verletzung

Die Definition „schwere Verletzung“ erfolgte in Vorarbeiten anhand der S3-Leitline Schwerverletzten- und Polytraumaversorgung [7], des NAIK [1], des AIS-Codebook (Abzug 2015) und der GIDAS-Variablen [8]. Neben Verletzungen des AIS-Schweregrades 3 (oder schwerer; AIS 3+) wurden ausgewählte GIDAS-Variablen als „Schwerverletzte“ definiert, da bei diesen eine Notarztindikation nach NAIK besteht. Eine Tabelle mit den GIDAS-Codes steht im Zusatzmaterial online zur Verfügung. Alle weiteren Personen wurden als „Leichtverletzte“ eingestuft.

### Statistik

Es wurden jeweils ein Datensatz für „Schwerverletzte“ und „Leichtverletzte“ erstellt und die relative Verteilung (in Prozent) der Unfallmodalitäten und deren Ausprägungen in beiden Datensätzen betrachtet und miteinander verglichen. Es folgte die Berechnung der Sensitivität (Sens.), Spezifität (Spez.), des positiven (PPW) und negativen prädiktiven Werts (NPW) der identifizierten Variablen. Die Nullhypothese, also eine fehlende Beziehung zwischen Unfallvariablen und Verletzungsschwere, wurde mittels Chi-Quadrat-Test überprüft. Ab einem Signifikanzniveau von *p* < 0,05 wurde die Nullhypothese verworfen und von einer statistisch signifikanten Beziehung zwischen Unfallvariable und Verletzungsschwere ausgegangen. Der Freiheitsgrad liegt immer bei 1, da 2 Ausprägungen bezüglich der Verletzungsschwere (Leicht‑, Schwerverletzte) sowie 2 Ausprägungen der untersuchten Variable (zutreffend, nicht zutreffend) vorliegen. Additiv wurden jeweils 2 Variablen kombiniert und der PPW erhoben.

### Relevante Unfallvariablen

Sämtliche GIDAS-Variablen wurden zunächst nach Unfallmodalitäten gefiltert. Die über 200 identifizierten Unfallvariablen wurden auf Laien-Erhebbarkeit geprüft und eine Vorauswahl von 89 Variablen getroffen. Anhand der Häufigkeitsverteilungen konnten schließlich 28 GIDAS-Variablen identifiziert werden, welche mit schweren Verletzungen assoziiert sind.

Hierzu zählen das Herausschleudern eines Insassen, Insasse eingeklemmt, Helmverlust von Zweiradaufsassen, brennendes Fahrzeug, Probleme bei der Bergung (eingeklemmt, Fzg. verklemmt), Pkw (teilweise) zerrissen, Amnesie, VRU mitgeschliffen/überrollt, Scheibenbruch am Gegnerfahrzeug, Folgeaufprall von Kraftradaufsassen mit Objekten, Intrusion des Pkw, Kollision mit „light goods vehicle“ (Nutzfahrzeug mit Gesamtgewicht bis 3,5 t, LGV) bzw. „heavy goods vehicle“ (Nutzfahrzeug mit Gesamtgewicht bis 40 t, HGV), fehlende Gurtnutzung, Unterfahren eines Pkw, Fzg. (Krad/Pkw) neben Fahrbahn, Zusammenstoß mit entgegenkommenden Fzg., Anzahl der Kollisionen eines Beteiligten ≥ 3, Scheibenbruch (bei eigenem Pkw), außerorts oder Bundesautobahn (BAB), Objektkollision, Pkw überschlagen, Überholmanöver auf Gegenfahrspur, Unfallzeit: nachts und Airbagauslösung. Insbesondere für die Kombinationsuntersuchung zwischen diesen Variablen wurden zudem die 4 Anprallrichtungen am Fahrzeug, Impact: frontal (Frontalzusammenstoß), Impact: „near side“ (Einschlag auf der dem Betroffenen zugewandten Fahrzeugseite), Impact: „far side“ (Einschlag auf der dem Betroffenen abgewandten Seite) und Impact: Heck (Heckaufprall) ergänzt. Die Liste der GIDAS-Variablen steht im Zusatzmaterial online zur Verfügung.

Bei Mehrfachkollisionen wurden bei Pkw-Insassen die schwerste (hinsichtlich Verletzungsfolge), bei zu Fuß Gehenden die initiale und bei Fahrrad- und Kraftradfahrenden ebenfalls die initiale Kollision betrachtet, sofern nicht eine Objektkollision ursächlich für die Kollision mit einem anderen Verkehrsteilnehmer war.

Da einige der Variablen nur auf bestimmte Arten der Verkehrsteilnahme anwendbar sind, erfolgte die Unterteilung in die Kategorien Fahrzeuginsassen (Fzg.-Insassen), Rad- oder Kraftradfahrende (Rad, Krad), und zu Fuß Gehenden (FG). Ungeschützte Verkehrsteilnehmer („vulnerable road user“, VRU) umfassen Rad, Krad und FG sowie Elektrokleinstfahrzeuge (EKF, beispielsweise E‑Scooter).

## Ergebnisse

**Figure 1:**
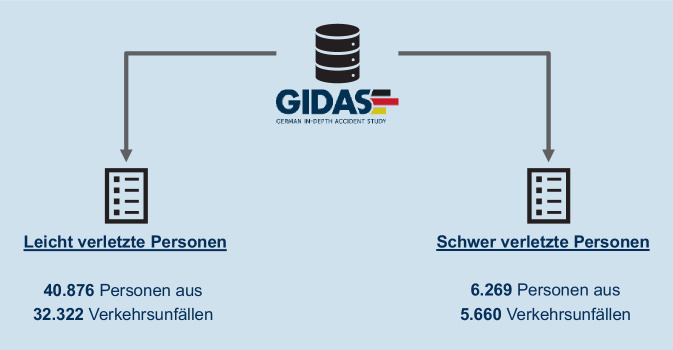

Im Erhebungszeitraum wurden 47.145 verunfallte Personen in die GIDAS-Datenbank eingeschlossen. Hiervon waren 40.876 (86,7 %) Personen leicht (AIS 1–2) und 6269 Personen (13,7 %) schwer verletzt (AIS 3+; Abb. 1). Personen ohne Verletzungsfolgen (AIS 0) nach einem Verkehrsunfall wurden nicht betrachtet.

Von den Betroffenen waren 20.447 (43,4 %) weiblich und 26.595 (56,4 %) männlich (Tab. 1), von 103 Verletzten lag kein dokumentiertes Geschlecht vor („unbekannt“). Männliche Verkehrsteilnehmer waren häufiger in Verkehrsunfälle mit schweren Verletzungen (64,2 %) verwickelt. Das mediane Alter betrug 36 Jahre (IQR 30 Jahre), Schwerverletzte waren mit einem medianen Alter von 42 Jahren (IQR 35 Jahre) älter als Leichtverletzte (medianes Alter 35 Jahre, IQR 29 Jahre).

**Table 1:** 

	Geschlecht			Alter (Jahre)		
	Männlich *n* (%)	Weiblich *n* (%)	Unbekannt *n* (%)	Minimum	Maximum	Median (IQR)
Verletzte	26.595 (56,41)	20.447 (43,37)	103 (0,22)	0	101	36 (23–53)
Davon leicht verletzt AIS 1–2	22.570 (55,22)	18.209 (44,55)	97 (0,24)	0	101	35 (23–52)
Davon schwer verletzt AIS 3+	4025 (64,2)	2238 (35,7)	6 (0,1)	0	97	42 (25–60)

Von den Verletzten waren 56,4 % männlich, bei den schweren Verletzungen (AIS3+) überwog der Anteil an männlichen Betroffen mit 64,2 % noch deutlicher. Das mediane Alter der Schwerverletzten lag mit 42 Jahren über dem Gesamtmedian von 36 Jahren

*Unbekannt* fehlende Dokumentation

Tab. 2 bildet die Verteilung der Betroffenen hinsichtlich der Art der Verkehrsteilnahme gegenüber der Verletzungsschwere ab. Von den 47.145 Betroffenen erlitten 6269 Personen schwere Verletzungen. Unfälle mit der Beteiligung vom Pkw (2756 Schwerverletzte), Radfahrenden (Rad) und Nutzenden von Elektrokleinstfahrzeugen (EKF, 1312 Schwerverletzte) führten, absolut betrachtet, am häufigsten zu schweren Verletzungsfolgen. Bei zu Fuß Gehenden (FG) und Kraftradfahrenden (Krad) wurden jeweils 1009 schwere Verletzungen erfasst, wobei der relative Anteil bei Verletzungsfolge unter zu Fuß gehenden Unfallpartnern mit 22 % überwog (verglichen mit 18,5 % bei Kraftradfahrenden).

**Table 2:** 

	Gesamter Datensatz *n* (%)	Pkw-Insassen *n* (%)	FG *n* (%)	Rad + EKF *n* (%)	Krad *n* (%)	Güter‑/Personentransport *n* (%)
Verletzte	47.145	23.759	4592	11.744	5458	1246
Davon leicht verletzt	40.876 (86,7)	21.003 (88,4)	3583 (78,0)	10.432 (88,8)	4449 (81,5)	1097 (88,0)
Davon schwer verletzt	6269 (13,3)	2756 (11,6)	1009 (22,0)	1312 (12,6)	1009 (18,5)	149 (12,0)

Pkw-Insassen machten mit 23.753 (50,4 %) den größten Anteil aus, gefolgt von Radfahrenden (Rad) und Elektrokleinstfahrzeugen (EKF) mit 11.744 (24,9 %). Von 6269 erfassten schwer verletzten Personen (AIS3+) waren 2756 Pkw-Insassen (11,6 % aller Pkw-Insassen), gefolgt von 1312 als Radfahrende und Nutzenden von EKF (12,6 % aller Radfahrenden/EKF-Nutzenden)

*FG* zu Fuß Gehende

Von den zahlreichen GIDAS-Variablen wurden insgesamt 28 als präklinisch, durch Laien erhebbar und damit durch einen Leitstellendisponenten abfragbar, eingestuft. Die Berechnungen der Gütekriterien zur Prädiktion einer schweren Verletzung sind zusammenfassend in Tab. 3 dargestellt.

**Table 3:** 

Verkehrsteilnahme	Variable	Betroffene n. Verkehrsteilnahme (in %)	Sens. (in %)	Sens. (nach Art Verkehrsteilnahme in %)	Spez. (nach Art Verkehrsteilnahme in %)	PPW (in %)	NPW (in %)	Chi-Quadrat	*p*
Fzg.-Insassen	*Herausgeschleudert*	0,6	2,1	4,4	99,9	*80,6*	88,8	746,8	*0,000*
Fzg.-Insassen	*Insasse eingeklemmt*	3,6	10,9	23,5	99,0	*75,6*	90,8	3731,9	*0,000*
Rad, Krad	*Helmverlust von Zweiradaufsassen*	0,8	1,3	3,5	99,7	*61,1*	86,9	256,0	*0,000*
Fzg.-Insassen, Krad	*Brennendes Fzg.*	0,6	1,5	2,5	99,7	*57,1*	87,4	296,0	*0,000*
Fzg.-Insassen	*Probleme bei Bergung (eingeklemmt, Fzg. verklemmt)*	6,9	15,5	33,4	96,6	*56,3*	91,7	3594,7	*0,000*
Fzg.-Insassen	*Pkw (teilweise) zerrissen*	0,8	1,6	3,4	99,6	*51,6*	88,7	300,7	*0,000*
Alle	*Amnesie*	6,9	25,9	25,9	96,1	*50,3*	89,4	4120,8	*0,000*
FG, Rad, Krad	*VRU mitgeschliffen/überrollt*	1,9	2,7	5,1	98,7	*41,9*	85,2	226,0	*0,000*
FG, Rad, Krad	*Scheibenbruch am Gegnerfahrzeug*	8,6	10,6	20,0	93,5	*35,8*	86,6	661,1	*0,000*
Krad	*Folgeaufprall mit Objekten von Kraftradaufsassen*	9,3	2,5	15,6	92,2	*31,1*	82,8	58,7	*0,000*
Fzg.-Insassen	*Intrusion des Pkw*	28,6	29,9	64,5	76,1	*26,2*	94,2	2073,3	*0,000*
Alle	*Kollision mit LGV/HGV*	6,4	11,9	11,9	94,5	*25,0*	87,5	377,9	*0,000*
Fzg.-Insassen	*Nicht angeschnallt*	6,8	6,2	13,4	94,1	*22,9*	89,2	224,6	*0,000*
Fzg.-Insassen	*Pkw, Unterfuhr*	1,9	1,7	3,6	98,4	*22,7*	88,6	56,2	*0,000*
Fzg.-Insassen, Krad	*Fzg. (Krad/Pkw) neben Fahrbahn*	6,9	7,3	11,7	94,1	*22,6*	87,8	184,9	*0,000*
Fzg.-Insassen, Rad, Krad	*Zusammenstoß mit entgegenkommenden Fzg.*	7,9	11,4	13,7	93,1	*21,9* *%*	88,4	301,9	*0,000*
Fzg.-Insassen, Krad	*Anzahl der Kollisionen eines Beteiligten ≥* *3*	10,4	10,3	16,5	90,9	*21,2*	88,1	211,5	*0,000*
Fzg.-Insassen	*Scheibenbruch (bei eigenem Pkw)*	38,8	32,4	69,9	65,3	*20,9*	94,3	1600,9	*0,000*
Alle	*Außerorts oder BAB*	25,6	40,1	40,1	76,6	*20,8*	89,3	792,8	*0,000*
Fzg.-Insassen, Krad	*Objektkollision*	26,4	25,2	40,4	77,0	*20,5*	89,8	545,5	*0,000*
Fzg.-Insassen	*Pkw, Überschlagen*	9,7	6,9	15,0	91,0	*18,0*	89,1	105,1	*0,000*
Alle	*Überholmanöver auf Gegenfahrspur*	1,7	2,1	2,1	98,4	*17,2*	86,8	10,6	*0,001*
Alle	*Nachts*	18,2	23,4	23,4	82,6	*17,1*	87,6	133,8	*0,000*
Fzg.-Insassen	*Airbagauslösung*	43,3	26,6	57,4	58,5	*15,4*	91,3	291,5	*0,000*
Fzg.-Insassen	*Impact: frontal*	43,9	25,3	54,5	57,5	*14,4*	90,6	151,8	*0,000*
Fzg.-Insassen	*Impact: „near side“*	15,7	8,6	18,6	84,7	*13,7*	88,8	20,0	*0,000*
Fzg.-Insassen	*Impact: „far side“*	11,1	6,0	12,9	89,2	*13,6*	88,6	12,0	*0,001*
Fzg.-Insassen	*Impact: Heck*	21,6	2,2	4,8	76,2	*2,6*	85,9	548,2	*0,000*

*Fzg.-Insasse* Fahrzeuginsasse, *Rad* Fahrradfahrende(r), *Krad* Kraftradfahrende(r), *FG* zu Fuß Gehende, *LGV* „light goods vehicle“ (Nutzfahrzeuge bis 3,5 t), *HGV* „heavy goods vehicle“ (Nutzfahrzeuge bis 40 t), *BAB* Bundesautobahn, *Impact: „near side“* Einschlag auf der dem Betroffenen zugewandten Fahrzeugseite, *Impact: „far side“* Einschlag auf der dem Betroffenen abgewandten Seite

Sortiert nach bester Vorhersagegüte anhand des PPW

Für Fahrzeuginsassen zeigte das Herausschleudern eines Insassen mit 80,6 % den höchsten PPW (Sensitivität 4,4 %, Spezifität 99,9 %, NPW 88,8 %), gefolgt von der Einklemmung eines Insassen mit 75,6 % (Sensitivität 23,5 %, Spezifität 99 %, NPW 90,8 %). Weitere, auf Pkw-Insassen anwendbare Variablen mit einem PPW von > 35 % sind brennendes Fahrzeug (PPW 47,1 %, Sensitivität 2,5 %, Spezifität 99,7 %, NPW 87,4 %), eine problematische Rettung der Betroffenen (PPW 56,3 %, Sensitivität 33,4 %, Spezifität 96,6 %, NPW 91,7 %), eine (teilweise) Zerreißung des Pkw (PPW 51,6 %, Sensitivität 3,4 %, Spezifität 99,6 %, NPW 88,7 %) und eine bestehende Amnesie der Betroffenen als Unfallfolge (PPW 50,3 %, Sensitivität 25,9 %, Spezifität 96,1 %, NPW 89,4 %).

Für Rad- oder Kraftradfahrende wiesen folgende Variablen einen PPW über 35 % für das Auftreten schwerer Verletzungen auf: Helmverlust (PPW 61,1 %, Sensitivität 3,5 %, Spezifität 99,7 %, NPW 86,9 %), brennendes Fahrzeug, Amnesie, VRU (fasst FG, Rad- und Kradfahrende zusammen) überrollt/mitgeschliffen (PPW 41,9 %, Sensitivität 5,1 %, Spezifität 98,7 %, NPW 85,2 %) und Scheibenbruch am Gegnerfahrzeug (PPW 35,8 %, Sensitivität 20 %, Spezifität 93,5 %, NPW 86,6 %).

Auf FG anwendbare Variablen mit PPW > 35 % sind Amnesie, VRU mitgeschliffen/überrollt und Scheibenbruch am Gegnerfahrzeug. Nach Anwendung des Chi-Quadrat-Tests weisen alle Unfallvariablen *p*-Werte < 0,05 und damit einen statisch signifikanten Zusammenhang mit der Verletzungsschwere auf.

### Optimierung der Vorhersage von schwerer Verletzung durch Kombination von Unfallvariablen

Durch die Kombination mehrerer Variablen können die PPW zur Prädiktion einer schweren Verletzung (AIS 3+) zusätzlich erhöht werden. Beispielsweise zeigt die Kombination aus Kollision mit LGV/HGV (PPW 25 % in der Einzelbetrachtung) und ungeschützter Verkehrsteilnehmer (VRU) überrollt/mitgeschliffen (PPW 41,9 % in der Einzelbetrachtung) einen Kombinations-PPW von 82,2 % (anwendbar auf FG, Rad, Krad). Weitere Kombinationen mit einem PPW > 60 % sind Scheibenbruch am Gegnerfahrzeug und Endlage des Fahrzeugs neben der Fahrbahn (PPW 80 %, anwendbar auf Rad und Krad), Unfallgeschehen außerorts oder auf BAB und VRU mitgeschliffen/überrollt (PPW 79,3 %, anwendbar auf FG, Rad, Krad), Unfallgeschehen außerorts oder auf BAB und gegnerisches Fahrzeug mit Scheibenbruch (PPW 64,9 %, anwendbar auf FG, Rad, Krad), Zusammenstoß mit entgegenkommendem Fahrzeug und Folgeaufprall mit einem Objekt (PPW 64,2 %, anwendbar auf Krad), VRU mitgeschliffen/überrollt und gegnerisches Fahrzeug mit Scheibenbruch (PPW 63 %, anwendbar auf Rad, Krad, FG). Weitere Kombinationen sind in Tab. 4 dargestellt.

**Table 4:** 

Variable 1	Variable 2	Sens. (in %)	Sens. (nach Art Verkehrsteilnahme in %)	PPW (in %)
*Kollision mit LGV/HGV*	*VRU mitgeschliffen/überrollt*	1,7	3,2	*82,2*
*Gegnerisches Fahrzeug mit Scheibenbruch*	*Endlage Fzg. neben Fahrbahn*	0,3	1,6	*80,0*
*Außerorts/BAB*	*VRU mitgeschliffen/überrollt*	0,4	0,7	*79,3*
*Außerorts/BAB*	*Gegnerisches Fahrzeug mit Scheibenbruch*	2,2	4,1	*64,9*
*Zusammenstoß mit entgegenk. Fzg*	*Folgeaufprall mit Objekt von Kraftradaufsassen*	0,3	1,8	*64,3*
*VRU mitgeschliffen/überrollt*	*Gegnerisches Fahrzeug mit Scheibenbruch*	0,3	0,5	*63,0*
*Außerorts/BAB*	*Folgeaufprall mit Objekt von Kraftradaufsassen*	1,2	7,3	*59,7*
*Endlage Fzg. neben Fahrbahn*	*Folgeaufprall mit Objekt von Kraftradaufsassen*	0,3	1,7	*56,7*
*Kollision mit LGV/HGV*	*Gegnerisches Fahrzeug mit Scheibenbruch*	0,5	1,0	*54,8*
*Anzahl der Kollisionen eines Beteiligten ≥* *3*	*Folgeaufprall mit Objekt von Kraftradaufsassen*	0,2	1,5	*53,6*
*Kollision mit LGV/HGV*	*Folgeaufprall mit Objekt von Kraftradaufsassen*	0,1	0,9	*50,0*
*Nicht angeschnallt*	*Überschlagen*	1,3	2,8	*48,5*
*Überholmanöver auf Gegenfahrspur*	*Folgeaufprall mit Objekt von Kraftradaufsassen*	0,1	0,8	*47,1*
*Zusammenstoß mit entgegenk. Fzg.*	*Unterfahrendes Fzg.*	0,3	0,7	*46,7*
*Anzahl der Kollisionen eines Beteiligten ≥* *3*	*Nicht angeschnallt*	1,7	3,7	*45,1*
*Überholmanöver auf Gegenfahrspur*	*Gegnerisches Fahrzeug mit Scheibenbruch*	0,2	0,5	*44,1*
*Nachts*	*Gegnerisches Fahrzeug mit Scheibenbruch*	3,0	5,7	*44,0*
*Objektkollision (motorisiertes Fzg.)*	*Folgeaufprall mit Objekt von Kraftradaufsassen*	0,9	5,5	*44,0*
*Zusammenstoß mit entgegenk. Fzg.*	*Gegnerisches Fahrzeug mit Scheibenbruch*	0,6	1,6	*43,0*
*Nicht angeschnallt*	*Intrusion des Pkw*	4,4	9,5	*42,0*
*Nachts*	*VRU mitgeschliffen/überrollt*	0,4	0,8	*41,2*
*Nachts*	*Folgeaufprall mit Objekt von Kraftradaufsassen*	0,4	2,7	*39,7*
*Kollision mit Güter‑/Personentransport*	*Zusammenstoß mit entgegenk. Fzg.*	1,5	1,8	*38,7*
*Zusammenstoß mit entgegenk. Fzg.*	*Intrusion des Pkw*	6,4	13,9	*38,3*
*Endlage Fzg. neben Fahrbahn*	*Nicht angeschnallt*	1,0	2,2	*37,6*
*Objektkollision (motorisiertes Fzg.)*	*Nicht angeschnallt*	3,6	7,8	*37,0*
*Unterfahrendes Fzg.*	*Intrusion des Pkw*	1,3	2,9	*35,8*
*Kollision mit LGV/HGV*	*Endlage Fzg. neben Fahrbahn*	0,5	0,8	*35,6*
*Außerorts/BAB*	*Nicht angeschnallt*	3,9	8,4	*35,1*
*Zusammenstoß mit entgegenk. Fzg.*	*Anzahl der Kollisionen eines Beteiligten ≥* *3*	0,8	1,3	*33,8*
*Zusammenstoß mit entgegenk. Fzg.*	*Endlage Fzg. neben Fahrbahn*	1,0	1,5	*33,7*
*Intrusion des Pkw*	*Impact: Frontal*	14,1	30,3	*33,4*
*Überholmanöver auf Gegenfahrspur*	*Anzahl der Kollisionen eines Beteiligten ≥* *3*	0,3	0,5	*33,3*
*Gegnerisches Fahrzeug mit Scheibenbruch*	*Folgeaufprall mit Objekt von Kraftradaufsassen*	0,3	1,9	*32,8*
*Kollision mit LGV/HGV*	*Unterfahrendes Fzg*	1,3	2,8	*32,4*
*Zusammenstoß mit entgegenk. Fzg.*	*Überschlagen*	0,4	0,8	*32,4*
*Überholmanöver auf Gegenfahrspur*	*Intrusion des Pkw*	0,8	1,8	*32,1*
*Außerorts/BAB*	*Intrusion des Pkw*	22,6	48,8	*31,9*
*Zusammenstoß mit entgegenk. Fzg.*	*Scheibenbruch (bei eigenem Fzg.)*	7,2	15,6	*31,9* *%*
*Nachts*	*Kollision mit LGV/HGV*	2,6	2,6	*31,9*
*Überholmanöver auf Gegenfahrspur*	*Objektkollision (motorisiertes Fzg.)*	0,7	1,1	*31,9*
*Nicht angeschnallt*	*Scheibenbruch (bei eigenem Fzg.)*	4,9	10,6	*31,4*
*Kollision mit LGV/HGV*	*Intrusion des Pkw*	5,3	11,5	*31,3*
*Außerorts/BAB*	*Zusammenstoß mit entgegenk. Fzg.*	7,4	8,8	*31,3*
*Zusammenstoß mit entgegenk. Fzg.*	*Nicht angeschnallt*	0,9	2,0	*31,2*
*Nicht angeschnallt*	*Impact: „far side“*	0,9	1,9	*30,5*
*Intrusion des Pkw*	*Airbagauslösung*	17,4	37,6	*30,3*
*Objektkollision (motorisiertes Fzg.)*	*Intrusion des Pkw*	14,6	31,5	*30,0*
*Nachts*	*Nicht angeschnallt*	2,4	5,2	*29,9*
*Endlage Fzg. neben Fahrbahn*	*Intrusion des Pkw*	3,7	7,9	*29,7*
*Außerorts/BAB*	*Kollision mit LGV/HGV*	5,1	5,1	*29,4*
*Nachts*	*Intrusion des Pkw*	8,8	18,9	*29,3*
*Kollision mit LGV/HGV*	*Nicht angeschnallt*	0,8	1,8	*28,7*
*Kollision mit LGV/HGV*	*Anzahl der Kollisionen eines Beteiligten ≥* *3*	0,9	1,5	*28,2*
*Intrusion des Pkw*	*Scheibenbruch (bei eigenem Fzg.)*	25,5	55,0	*28,1*

Gesamtsensitivität (Sens.), Sensitivität nach Art der Verkehrsteilnahme und des PPW von Kombinationen ausgewählter Einzelvariablen; sortiert nach bester Vorhersagegüte anhand des PPW

Auch Variablen mit geringen PPW in der Einzelbetrachtung, wie ein nächtliches Unfallereignis (Einzel-PPW 17,1 %), können in Kombination, beispielsweise Gegnerfahrzeug mit Scheibenbruch (Einzel-PPW 35,8 %) einen validen PPW zur Prädiktion einer schweren Verletzung generieren (am Bsp. Kombinations-PPW 44 %).

## Diskussion

Ziel dieser Arbeit ist die Überprüfung von NAIK-Kriterien anhand objektiver Daten der GIDAS-Datenbank zur evtl. Novellierung des aktuellen NAIK nach Verkehrsunfällen der Bundesärztekammer. Vor dem Hintergrund sich wandelnder Verkehrsinfrastruktur, verbesserter Verkehrssicherheitssysteme, steigender Einsatzzahlen des Rettungsdienstes und teilweise nichtsichergestellter notärztlicher Abdeckung können durch eine Novellierung der Notarztindikationen eine Optimierung der Rettungskette sowie ökonomische und bedarfsgerechte Alarmierungen von Notärzt*innen die Rate an Fehlalarmierung mutmaßlich reduziert und die Patientenversorgung verbessert werden.

Eine kurze präklinische Versorgungszeit [9] bzw. die adäquate Durchführung notwendiger (invasiver) präklinischer Maßnahmen zur Versorgung Schwerverletzter [10] können sich positiv auf das Outcome von Traumapatienten nach VKU auswirken.

Da die Erhebungsgebiete Dresden und Hannover topografisch den Bundesdurchschnitt repräsentieren, die Erhebung nach einem genau definierten Stichprobenplan erfolgt und die Fallzahl entsprechend hoch ist, kann die GIDAS-Datenbank aus Sicht der Autoren für repräsentative Aussagen zum deutschen Unfallgeschehen genutzt werden [11, 12]. Ziel des gewählten Erhebungszeitraums war, den aktuellen Stand der Fahrzeugtechnik widerzuspiegeln. Valide Unfallvariablen wurden identifiziert, welche auf schwere Verletzungen (AIS 3+) als Unfallfolge schließen lassen und präklinisch durch Laien erhebbar sind. Mit der Definition des Schweregrades AIS 3+ als „schwere Verletzungen“ wird ebenfalls das Schutzziel der Automobilindustrie, mit dem Ziel der Vermeidung ebendieser Unfallfolgen, abgebildet.

Ferner werden vital bedrohliche Verletzungen und schwere anatomische Verletzungen mit Schmerzen und mutmaßlichem Blutverlust (z. B. Fraktur großer Röhrenknochen) abgebildet.

Über 200 GIDAS-Variablen (Zusatzmaterial online), welche technische, geografische und zeitliche Unfallmodalitäten beschreiben, wurden nach präklinischer Erhebbarkeit durch Laien gescreent. 89 dieser Variablen erscheinen prinzipiell durch Laien erhebbar, weisen allerdings in der Einzelbetrachtung kein erhöhtes Aufkommen im Datensatz „schwer verletzte Personen“ auf bzw. sind im Rahmen eines akuten Unfallereignisses nicht fassbar. Ggf. wäre hier die Übernahme in einen Fragenkatalog für Rettungsleitstellendisponenten möglich, um durch Mehrfachkombination dieser Parameter schwere Verletzungen vorherzusagen. Als Einzel- oder Kombinationsvariable waren 28 Unfallmodalitäten mit schweren Verletzungen als Unfallfolge assoziiert.

### Nachteile des aktuellen NAIK

Die im aktuellen NAIK aufgeführten Änderungen der Vitalfunktionen, welche zu einer Alarmierung führen, beispielsweise Schädel-Hirn-Trauma, Thorax‑/Beckentrauma, Frakturen mit deutlichen Fehlstellungen, Schmerzen, sind für Laien kaum und durch nichtärztliches Rettungsdienstpersonal erst nach Rettung und Untersuchung des Patienten feststellbar und somit für die Bewertung eines Notrufs und Alarmierung eines Notarztes nur bedingt geeignet. Die aktuellen Kriterien sind durch nichtärztliches Rettungsdienstpersonal erhebbar, würden jedoch zu einer Nachforderung eines Notarztes an die Unfallstelle mit relevantem Zeitverlust führen. Deshalb empfehlen die Autoren, diese Kriterien aus dem NAIK zu entfernen.

Auch die Formulierungen „Bei Verdacht auf fehlende oder deutlich beeinträchtigte Vitalfunktion“, nach „schwerem Verkehrsunfall mit Hinweis auf Verletzte“ bzw. „sonstiger Unfall mit Schwerverletzten“ liefern keine klaren Kriterien, welche zu einer frühzeitigen Notarztalarmierung führen sollten und lassen so viel Interpretationsspielraum für die Rettungsleitstellendisponenten.

### Empfehlung

Die Integration der präklinisch durch Laien festzustellenden Unfallmodalitäten in den NAIK scheint naheliegend, da die identifizierten Variablen bereits beim Eintreffen an der Unfallstelle oder mittels Abfrage durch Disponent*innen festgestellt werden können.

Für Fahrzeuginsassen sind dies:

- Das Herausschleudern eines Insassen
- Einklemmung eines Insassen
- Brennendes Fahrzeug
- Eine problematische Rettung der Betroffenen
- Die (teilweise) Zerreißung des Pkw und
- Eine bestehende Amnesie der Betroffenen als Unfallfolge.

Für Rad- oder Krad-Fahrende weisen

- Helmverlust
- Brennendes Fahrzeug
- Amnesie
- VRU überrollt/mitgeschliffen und
- Scheibenbruch am Gegnerfahrzeug

hohe PPW für schwere Verletzungen auf.

Auf FG anwendbare Variablen sind:

- Amnesie
- VRU mitgeschliffen/überrollt und
- Scheibenbruch am Gegnerfahrzeug.

Diese sollten mit einem PPW für schwere Verletzungen als Einzelvariable von > 35 % in den NAIK aufgenommen werden.

Auch Kombinationsvariablen, beispielsweise ein Scheibenbruch am Gegnerfahrzeug und Endlage des Fahrzeugs neben der Fahrbahn oder ein Scheibenbruch am Gegnerfahrzeug mit Unfallort außerorts/auf BAB, wiesen signifikante Assoziationen mit schweren Verletzungen für Kraftradfahrende auf.

Die errechneten Signifikanzniveau-Werte unterstreichen die Assoziation zwischen den identifizierten Variablen und dem Auftreten schwerer Verletzungen. Die geringen *p*-Werte sind mit der Vorauswahl der Variablen anhand der relativen Häufigkeiten im Datensatz zu erklären.

Einhergehend mit der Schockraumindikation „Herausschleudern eines Insassen“ bzw. der bereits im NAIK enthaltenen Indikation „Brände/Rauchgasentwicklung mit Hinweis auf Personenbeteiligung“ sowie „Einklemmung oder Verschüttung“ empfehlen wir die Aufnahme folgender konkreter Unfallmodalitäten als Notarztindikation: *Herausschleudern eines Insassen, brennendes Fahrzeug, problematische Rettung durch Verklemmung der Person oder des Fahrzeugs, Helmverlust von Zweiradaufsassen, Pkw (teilweise) zerrissen, Amnesie eines Betroffenen, VRU mitgeschliffen/überrollt und Scheibenbruch am Gegnerfahrzeug*.

### Limitationen

Einschränkend ist die präklinische Beurteilbarkeit mancher der dargelegten Variablen. Beispielsweise ist es für Laien nicht immer möglich, Amnesie, Mehrfach- oder Objektkollisionen festzustellen. Dies könnte jedoch nach Schulung des Rettungsleitstellenpersonals zielsicher abgefragt werden. Hierbei hat sich in einem parallelen Projekt unserer Arbeitsgruppe zur Optimierung des E‑Call ein deutlicher Lerneffekt in der korrekten Einschätzung von Verkehrsunfällen und deren Schwere gezeigt.

Ferner wurden schwere Verletzungen nach Definition der Arbeitsgruppe als Notarztindikation angesehen. Eine Übertriagierung aufgrund von beispielsweise Minoramputationen (beispielsweise Finger, Zehen) oder geschlossenen Frakturen langer Röhrenknochen ist daher nicht auszuschließen. Die Notwendigkeit einer entsprechenden i.v.-Analgesie und Reposition relativiert dies jedoch.

## Fazit für die Praxis

Eine Reevaluation der Parameter und v. a. des Automobil-Schutzziels von AIS3+ in der Zukunft ist aus Sicht der Autoren sinnvoll und sollte zur Optimierung der Notarztindikationen nach Verkehrsunfällen erfolgen. Zudem ist eine Überarbeitung bei grundlegenden Innovationen der Fahrzeugsicherheitstechnik empfehlenswert.

In prospektiven Analysen sollte die Anwendung der Unfallvariablen verifiziert werden. Ggf. ergeben sich daraus ebenfalls die Notwendigkeit der Reevaluation des Schutzziels der Automobilindustrie und damit die Definition der schweren Verletzung. Anhand der gewonnenen Daten könnte ein Fragenkatalog für Rettungsleitstellendisponent*innen entworfen werden, welcher sich an Ersteintreffende bei Verkehrsunfällen richtet und Kombinationen möglicher Unfallparameter abfragt. Schwere Verletzungen könnten so anhand valider Daten identifiziert und damit bedarfsgerechte Primäralarmierungen ermöglicht werden, um Zeitversäumnisse in der präklinischen Schwerverletztenversorgung zu vermeiden und so das Outcome der Patienten zu verbessern. Auch eine Erfassung weiterer Unfallparameter zur Verletzungsvorhersage nach Verkehrsunfall ist ein Anwendungsbeispiel, welches diese Projektgruppe bereits bearbeitet, um automatisierte Notrufe von verunfallten Fahrzeugen an alle Mitglieder der Rettungskette zu senden.

Basierend auf Registerdaten wurden objektive Variablen zur präklinischen Vorhersage schwerer Verletzungen nach Verkehrsunfällen identifiziert. Im Kontext steigender Alarmierungszahlen sollten alte Indikationsvariablen zur Notarztalarmierung nach Verkehrsunfall verworfen und neue Variablen in den neuen Notarztindikationskatalog der BÄK aufgenommen werden:

- Reevaluation Notarztindikationen für Verkehrsunfälle bei grundlegenden Innovationen der Fahrzeugsicherheitstechnik empfehlenswert
- Identifizierung Variablen zur präklinischen Vorhersage schwerer Verletzungen: Herausschleudern eines Insassen, brennendes Fahrzeug, problematische Rettung durch Verklemmung der Person oder des Fahrzeugs, Helmverlust von Zweiradaufsassen, Pkw (teilweise) zerrissen, Amnesie eines Betroffenen, VRU mitgeschliffen/überrollt, Scheibenbruch am Gegnerfahrzeug;
- Reevaluation des Automobilindustrieschutzziels und Definition schwerer Verletzungen auf Grundlage von Daten erforderlich
- Entwicklung eines Fragenkatalogs für Rettungsleitstellendisponent*innen zur Identifikation schwerer Verletzungen möglich
- Prospektive Analyse zur Verifizierung der Anwendung der Unfallvariablen notwendig
- Nutzung zusätzlicher Unfallparameter zur Verletzungsvorhersage und automatisierten Notrufsendung technisch möglich (E‑Call).

### Supplementary Information




