# Supplementary material for: Reassessment of the emergency medical service deployment indication catalogue after traffic accidents
Source: Unfallchirurgie (Heidelb). 2024 Feb 2;127(5):364–73. [Article in German] doi: 10.1007/s00113-023-01408-8 (PMC11058606; doi:10.1007/s00113-023-01408-8)
Supplement: Supplementary file 1 [file 113_2023_1408_MOESM1_ESM.pdf]

Rekord	Variable	Bedeutung	Geeignet für Abfrage
**Allgemeine Daten des Unfalles**	TZEIT	Tageszeit	ja
	ORTSL	Unfallstelle innerhalb oder außerhalb einer geschlossenen Ortschaft	ja
	ANZBET	Anzahl Beteiligter	
	ANZBETFZ	Anzahl beteiligte Fahrzeuge	ja
	ANZPERS	Anzahl beteiligte Personen	ja
	ANZVERL	Anzahl verletzte Personen	ja
	HURSAB	Unfallverursacher amtlich	
	HURSAU	Haupt-Unfallursacher amtlich	
	FOLGE1 / 2	Folgeunfall	
	UTYP	Unfalltyp	
	UART	Unfallart	
	TEMP	Temperatur	
	WINDV / WINDS	Windverhalten	
	WOLK	Bewölkung/Nebel	ja
	NIED	Niederschlag	ja
	UMGEB	Umgebung	ja
	STFUHO	Unfallstelle im Straßennetz	
	TUNNEL	Tunnel	ja
	VKREG	Verkehrsregelung	
	VSTUFE	Verkehrsstufe	
**Unfallfahrzeug**	FART	Fahrzeugart	ja
	FZGB	Sichtbare Deformation am Fahrzeug	ja
	ANZINS	Anzahl der Insassen	ja
	FZART	Fahrzeugart (Aufbau)	ja
	ABF	Aufbauform	ja
	FZGKLASS	Fahrzeugklasse	ja
	KLASSECE	Fahrzeugklasse amtlich (gelabelt)	
	LSTG	Leistung in kw	
	LAENGE	Länge	
	BREITE	Breite	
	HOEHE	Höhe	
	LGEW	Leergewicht	
	GEWGES	Crashgewicht	
	BSPUR	Bremsspur vor Kollision	
	SSPUR	Drift- oder Schleuderspurzeichnung vor Kollision	
	BENZAUS	Kraftstoff ausgelaufen	
	OELAUS	Öl ausgelaufen	
	FLAUS1-3	Flüssigkeit ausgelaufen	
	BRANDURS	Ursprung des Feuers?	
	ENDLAGE	Endlage des Fahrzeuges (auf, neben, ..., der Fahrbahn)	ja
	ANHAENGER	Anhänger mitgeführt	ja
**Personendaten**	GESCHL	Geschlecht	ja
	GROESP / GROESPG	Körpergröße	evtl. clustern
	GEWP / GEWPG	Gewicht	evtl. clustern
	ALTER1 / ALTERG	Alter	evtl. clustern
	KISITZ	Kindersitz	ja
	UNVERMOG	Unvermögen	

**Verletzungsübersicht**	AMNES	Amnesie	evtl.
	PUPILLE	abnorme Pupillen	
**Einzelverletzung**	VTEIL1 / 2	Anprallstelle	
	AIS15REG	AIS-Schweregrad	
**Rettungskette**	BEWPERS	Bewusstseinslage Unfallort	ja
(nur präklinisch)	BERGM	Rettungs-/Bergungsmaßnahmen	evtl.
	TBERG1 - 3	techn. Rettung/Bergung	evtl.
	ERSTH	Erste Hilfe geleistet	ja
	NOTVER	Maßnahmen zur Blutstillung (Ersthelfer)	ja
	AWFREI	Atemwege befreien (Ersthelfer)	ja
	ATEMSP	Atemspende (Ersthelfer)	ja
	HERZMA	Herzmassage (Ersthelfer)	ja
	PATEIN	Lage Patient bei Eintreffen der Sanitäter	ja
	GCS1 - 3 / GCSG	GCS	
	UDRUCKV	Maßnahmen zur Blutstillung (Unfallort)	
	UBEATM	Beatmung am Unfallort	
	UINTUB	Intubation am Unfallort	
	UREA	Reanimation am Unfallort	
**Insassenbewegung**	EINGEKL	Insasse eingeklemmt	ja
	AUSSCHL	Insasse herausgeschleudert	ja
**PKW allgemein**	(LONGCM)	Deformation Längsträger (in cm)	
	(FLTCM)	Deformation über Längsträger hinaus (cm)	
**PKW Beschädigungen von außen**	FZGFB	Fzg-Front beschädigt	ja
(eigener PKW und / oder Kollisionsgegner)	FZGSLB	Seite links beschädigt	ja
	FZGSRB	Seite rechts beschädigt	ja
	FZGHB	Fzg-Heck beschädigt	ja
	(FELGVL / HL / VR / HR)	Felge beschädigt	ja
	(ACHSVL / HL / VR / HR)	Achse beschädigt	
**LKW allgemein**	USCHUTZ	Unterfahrschutz	
**LKW Beschädigungen von außen**	LFZGFB	Fzg-Front beschädigt	ja
(Kollisionsgegner)	LFZGSLB	Seite links beschädigt	ja
	LFZGSRB	Seite rechts beschädigt	ja
	LFZGHB	Fzg-Heck beschädigt	ja
**Zweirad allgemein**	-		
**Fahrzeuginnenraum**	-		
**Technische Daten des Anhängers**	-		
**Fahrzeugbild außen**	ZRISS	Fahrzeug zerrissen	ja
(eigener PKW und / oder Kollisionsgegner)	MAXDEFB	Maximale Deformation (in cm)	evtl. clustern
	FSBRUCH1 / 2	1. Beschädigung Frontscheibe	ja
	HSBRUCH1 / 2	1. Beschädigung Heckscheibe	ja
	LONGDEF	Deformation Längsträger	
	TUERZVL	Zustand Tür vorn links	
	TVLINTR	Intrusion Tür vorn links	ja
	TUERZVR	Zustand Tür vorn rechts	
	TVRINTR	Intrusion Tür vorn rechts	ja
	TUERZHL	Zustand Tür hinten links	
	THLINTR	Intrusion Tür hinten links	ja
	TUERZHR	Zustand Tür hinten rechts	

	THRINTR	Intrusion Tür hinten rechts	ja
**Fahrzeugbild innen**	DEFIN	Innenraum verändert	
**Daten der Befragung eines Unfallbeteiligten**	-		
**Fußgänger**	VERHFUSSG	Verhalten Fußg. vorm Anprall	
	FGVKOL	Geschwindigkeit Fußgänger bei Kollision	
	UQFB	Überqueren der Fahrbahn	
	UQFB2	Überqueren der Fahrbahn zwischen	
	SPORT	benutztes Mobilitätsmittel	ja
	FGUART	Art der Fußgänger-Kollision	
	FGKIN	Fußgängerkinematik	
	FGRI	Richtung Fußgänger zu Fahrzeug	
	FGANST	Anstoßstelle am Fußgänger	
	FGUBER	Überrollen / Überfahren des Fußgängers	ja
	WWEITFG	Wurfweite Fußgänger (in cm)	evtl. clustern
**Zweiradaufsasse**	KINGRP	Bewegungsablauf des Zweiradaufsassen (nicht des Zweirads!) während der Kollisionsphase	
	AUSGRP	Mit der Auslaufgruppe wird der Bewegungsablauf des Zweiradaufsassen nach der Kollisionsphase der Primärkollision beschrieben	
	LKOMBI1 / 2	Kombi ein-/zweiteilig	ja
	LSTIEF	Stiefel	ja
	WWZWF	Wurfweite Zweiradaufsasse (in cm)	evtl. clustern
	PROT S / OA / E / UA / H / R / T /OS / K / US	Protektor	ja
	ZWHELM	Helmart	
	HVERL	Helmverlust	ja
	KINNR / KINNRH	Kinnriemen	
	VISIERZ	Zustand von Visier, Brille	ja
**Rekonstruktionsdaten**	KONOBJ	Kollisionskontrahent - Objekt	ja
(eigener PKW und / oder Kollisionsgegner)	V0	Ausgangsgeschwindigkeit	
	EPSILON	Stoßfaktor	
	VK	Geschw. Sequenzende / Koll.geschwindigkeit	
	BV	Mittlere Bremsverzögerung im Vorgang	
	DWINK	Ablenkungswinkel (°)	
	KWINK	Kollisionswinkel (°)	
	IMP	Impulswinkel	
	EES	Energy Equivalent Speed	
	DV	Delta-v (in km/h)	
	VREL	Relativgeschwindigkeit	
	DECK / SCHDECK	Überdeckungsgrad (in %)	
	ABKOM	Abkommen von Fahrbahn	ja
	(VDI1-7)	prinzipielle Richtung der Kraft, die den vorliegenden Schaden an dem Fahrzeug verursacht hat	
	PCCDC	Insassenzelle deformiert	ja
	UFAHR + ARTUFAHR	Unterfahren	ja
	UFAHRCM	Unterfahren, Länge (in cm)	evtl. clustern
	PLANKEK	Kollisionsart mit Schutzplanke	ja
**Sitzdaten**	RHSBEN	Gurt benutzt	ja
	AIRBF / SI / TI / DI / KN	Airbag Auslösung	ja
**Verkehrsunfallanzeige**	-		

**Mitfahrer laut VU Anzeige**	-		
**Beteiligte laut VU Anzeige**	-		
**Fahrzeugausstattung**	AIRBVRUA	Aktivierung des Fußgänger-/Radfahrer-Airbags	ja
(eigener PKW und / oder Kollisionsgegner)	POPVRUA	Aktivierung der Pop-up-Haube	ja
	BREMSASS	Bremsassistent	
	AEBLANG	AEB/Notbremssystem Längsverkehr	
	AEBQUER	AEB/Notbremssystem Querverkehr	
	AEBFGRF	AEB/Notbremssystem Fußgänger/Radfahrer	
	PRECRASH	Vorkonditionierung von Sicherheitssystemen	
**Daten des Unfallbeteiligten**	ARTTEIL	Art der Verkehrsteilnahme	ja
**Daten der befahrenen Straße**	RICHT	Richtung vor Unfall	ja
	STRDECK	Straßendecke	
	STROB	Straßenoberfläche	ja
	FSTREIF	Fahrzeug befuhr	
	UEBERHOL	Überholen	
	RICHTVU	Fahrlinie vor Unfall	
	RICHTUE	Straßenführung bei Unfall	
	FSPUR	Anzahl Fahrstreifen	ja
	FSPURG	Anzahl Fahrstreifen in Gegenrichtung	ja
	BAUST	Baumaßnahmen	ja
	AQUAPL	Aquaplaning-Einfluss	
	VZUL	zulässige Höchstgeschwindigkeit	ja
	UVERBOT	Überholverbot	ja
**Allgemeine Daten der Rekonstruktion**	ANZKOLL	Anzahl der Kollisionen	
	ROLLWANN	Überschlag	ja
	SCHLEU	Instabiler Fahrzustand vor Unfall	
	SCHLEU2	Fahrzustand bei Unfalleinleitung	
**Einzelanpralldaten**	ANTEIL	Anprallstelle am Fahrzeug	
	ANART	Anprallart	
	ANKT	Angepralltes Körperteil	
**Fahrrad allgemein**	-		
**Fahrrad Beschädigungen**	FRBESCH	Fahrrad beschädigt	ja
	FRROLL	Fahrrad überrollt	
	FRRAHMEN	Rahmenbeschädigung	ja
	FRFELGV	Felge vorn	ja
	FRFELGH	Felge hinten	ja
**Motorisiertes Zweirad allgemein**	-		
**Motorisiertes Zweirad Beschädigungen**	MZBESCH	motor. Zweirad beschädigt	ja
	MZROLL	motor. Zweirad überrollt	
	MZFELGV	Felge vorn	ja
	MZFELGH	Felge hinten	ja
	MZRAHMEN	Rahmenbeschädigung	ja
**Kinder-Rückhaltesystem**	-		
**Reifendaten**	-		
**PKW Innenraum**	IDAMAGE	Beschädigungen innen	
(seit 2017 + kaum durch Laie erhebbar)	IMIRROR	Innenspiegel	
	IAPILLTL	A-Säule oben links inkl. Verkleidung	
	ISTWHEEL	Lenkrad, Lenkradkranz	
	ISTWHSPK	Lenkrad, Speichen inkl. Bedienelemente	

	IPEDALTHR	Pedalerie, Gaspedal	
	IPEDALBRK	Pedalerie, Bremspedal	
	IPEDALCL	Pedalerie, Kupplungspedal	
	IAPILLTR	A-Säule oben rechts inkl. Verkleidung	
	IBPILLTL	B-Säule oben links inkl. Verkleidung	
	ICPILLTL	C-Säule oben links inkl. Verkleidung	
	IDPILLTL	D-Säule oben links inkl. Verkleidung	
	IBPILLTR	B-Säule oben rechts inkl. Verkleidung	
	ICPILLTR	C-Säule oben rechts inkl. Verkleidung	
	IDPILLTR	D-Säule oben rechts inkl. Verkleidung	
**LKW Innenraum**	ITDAMAGE	Beschädigungen innen	
**Bus allgemein**	BUSCHUTZ	Unterfahrschutz	
**Schienenfahrzeug allgemein**	-		
**Andere Nutzfahrzeuge allgemein**	NUSCHUTZ	Unterfahrschutz	
(bei Kollisionsgegner)	NFZGFB	Fzg-Front beschädigt	ja
	NFZGSLB	Seite links beschädigt	ja
	NFZGSRB	Seite rechts beschädigt	ja
	NFZGHB	Fzg-Heck beschädigt	ja
**Bus Beschädigungen außen**	BFZGFB	Fzg-Front beschädigt	ja
(bei Kollisionsgegner)	BFZGSLB	Seite links beschädigt	ja
	BFZGSRB	Seite rechts beschädigt	ja
	BFZGHB	Fzg-Heck beschädigt	ja
**ElektroKleinstfahrzeug**	ESCHADV	Beschädigungen vorn	ja
	ESCHADH	Beschädigungen hinten	ja
	ESCHADR	Beschädigungen rechts	ja
	ESCHADL	Beschädigungen links	ja
